# Individual patient data meta-analysis: a cost-effective and efficient tool to advance paediatric research in low- and middle-income countries

**DOI:** 10.1136/bmjopen-2025-100150

**Published:** 2026-05-13

**Authors:** Frederick McElwee, Andrew Farlow, Philippe J. Guerin

**Affiliations:** 1 Economics of Population Health Research Centre, University of Oxford, Oxford, England, UK; 2 Nuffield Department of Medicine, University of Oxford, Oxford, England, UK; 3 WorldWide Antimalarial Resistance Network (WWARN), Oxford, England, UK; 4 Infectious Diseases Data Observatory (IDDO), Oxford, England, UK; 5 Centre for Tropical Medicine and Global Health, Nuffield Department of Medicine, University of Oxford, Oxford, England, UK

**Keywords:** Decision Making, HEALTH ECONOMICS, Tropical medicine, Meta-Analysis, Neglected Diseases, Research Design

## Abstract

There is a deficit between existing levels of paediatric clinical research in low- and middle-income countries (LMICs) and what is warranted based on the disease burden in children in these countries. Lack of market protections, low ability to pay and cost-based pricing are key commercial barriers to industry-funded paediatric research and access to paediatric formulations of therapeutics in LMICs. Individual patient data meta-analysis (IPD-MA) taking advantage of existing clinical trials, enabled by data sharing, is one potential tool to bridge the research gap in a cost-effective and efficient way. Recent IPD-MAs of paediatric antimalarial drug safety and efficacy enabled by the WorldWide Antimalarial Resistance Network (WWARN), part of the Infectious Diseases Data Observatory (IDDO), demonstrate the feasibility of this approach.

## Introduction

 There is a deficit between existing levels of paediatric clinical research in low- and middle-income countries (LMICs) and what is warranted based on the disease burden in children in these countries.^1^ This shortfall is attributable to various ethical, practical and economic barriers^2 3^ and it persists despite regulatory efforts in high-income countries (HICs) to encourage or mandate clinical trials of new drugs in paediatric populations. The resultant lack of clinical trial evidence means that medicines are prescribed to children off-label or unlicensed.^2^ Dosing guidelines and/or available formulations may not cater to these off-label uses despite the importance of paediatric-specific dosing and formulations.^4^ Without directly relevant trial evidence, dosing in children is based on extrapolating from guidelines produced based on studies conducted in (disproportionately male) adult populations, adjusting for likely relevant factors such as body weight. However, pharmacological responses to medicines can differ between adults and children even after these adjustments, making such extrapolations potentially suboptimal.^2 5 6^ The heterogeneity of paediatrics—from neonates to adolescents—necessitates additional granularity and customisation of dosing guidelines and formulations.^6^ Children in LMICs would benefit from better dosing guidelines and access to paediatric formulations.

## Commercial drivers of the paediatric drug R&D shortfall

The commercial landscape for medicines in LMICs is challenging, especially for paediatric formulations, often leading developers to prioritise investments in other areas.^5^ Weak intellectual-property and market-exclusivity protections can deter investment by hindering the ability of companies to recoup the costs of paediatric drug research and development (R&D). Additional contributing factors include limited market size, low ability to pay for medicines and cost-based approaches to pricing, which mean that those funding research leading to or demonstrating improved effectiveness—for example, via better dosing guidelines—are unlikely to be rewarded with a higher price. These economic impediments contribute to insufficient market incentives for industry sponsors to fund further studies that have sufficient statistical power to change guidelines and secure label expansions, thereby perpetuating gaps in knowledge and treatment options for paediatric populations in LMICs. These factors, together with barriers to the development, distribution and affordable access to paediatric formulations, mean that all too often children fail to receive the right dose of the right medicine at the right time. In particular, even if children might benefit from a better dosing regimen, it would generate no financial reward for a company and not justify to them the costs of a clinical trial. We review below two cases of antimalarials where these commercial factors impeded paediatric R&D.

## Bridging the gap with push, pull and ‘pulley’ mechanisms

In HICs, postmarket commitments or requirements have been established to foster paediatric research and to develop paediatric indications. However, because the burdens of diseases such as malaria, HIV and tuberculosis (TB) differ between LMICs and HICs, such commitments cannot be relied on to finance paediatric drug R&D that would primarily benefit LMICs.^7 8^

Both government and philanthropic funders have sought to fill this gap with a variety of push and pull incentives that support investment in paediatric research and strengthen the market for paediatric formulations of essential medicines. Push mechanisms are ‘supply-side’ incentives which aim to reduce the costs and risks associated with drug R&D. These can take the form of research grants and tax credits. In global public health, a commonly employed push mechanism has been the formation of collaborative ventures, often known as Product Development Partnerships, in which participating stakeholders pool resources, knowledge and investment risk in the research and development of drugs. Examples include the Medicines for Malaria Venture (MMV), Drugs for Neglected Diseases *Initiative* (DND*i*), Medicines Development for Global Health, Global Antibiotic Research and Development Partnership, Combating Antibiotic-Resistant Bacteria Biopharmaceutical Accelerator and the TB Alliance.

Likewise, pull mechanisms—such as advanced market commitments and subsidies like the ‘Affordable Medicines Facility-malaria’—can improve the commercial environment for developers by providing economic incentives to scale up manufacturing capacity and sometimes the development of new medicines and formulations.^8^

Once medicines are developed with the proper formulations and clinical guidelines, it is vital that their benefits are realised by providing affordable and reliable access to patients in need. Organisations like UNITAID, MMV, DND*i*, TB Alliance and the Clinton Health Access Initiative have supported efforts to provide affordable access in LMICs to medicines such as antiretrovirals and treatments for multidrug-resistant TB, including special formulations for very young children.^9^,^11^ In addition, the Global Accelerator for Paediatric Formulations Network, a network hosted by the WHO, convenes and coordinates stakeholders to encourage development in this area. This network has identified several high-priority areas needing additional attention to develop paediatric oral dosage forms, such as acoziborole for human African trypanosomiasis and miltefosine and amphotericin B for visceral leishmaniasis, where commercial incentives are weak and trials hard to fund.^12^

However, in many countries, there is still too long a delay between when medicines first become available in adult dosages and when paediatric formulations are offered and can be administered using dose guidelines that are based on rigorous paediatric-specific evidence. For example, artemether–lumefantrine is a fixed-dose ACT indicated for uncomplicated *Plasmodium falciparum* malaria that was first registered in 1999 as a tablet formulation.^13^ To administer doses appropriate for infants and small children, the tablet must be crushed, but this is often poorly tolerated due to bad taste (especially of quinoline derivatives) and can cause drug loss, increasing the risk of subtherapeutic dosing.^13 14^ Yet, a dispersible paediatric formulation was not registered until nearly 10 years after initial registration, with WHO prequalification of this reformulation not occurring until 2009.^13 14^ Similarly, a formulation for neonates and young infants of the same drug was registered 26 years after its initial approval and prequalified by WHO 27 years after first registration.^11 15^

Innovative approaches are needed to further close the gap between current levels of paediatric research and what is warranted based on global health needs.^16^ Given the limitations on available resources, one approach is to embrace more efficient research methods that do more with less. We propose that funders should look beyond ‘push’ and ‘pull’ mechanisms and embrace new research models that can be conceptualised as ‘pulley mechanisms’—that is, models that have the potential to yield an outsized research impact with a relatively small investment or effort. Much like a pulley system can multiply a small force to lift a heavy weight, so a ‘pulley mechanism’ can leverage modest investments into significant benefits to global health by amplifying the impact of other research outputs.

## The role of data platforms as ‘pulley mechanisms’: generating evidence using IPD meta-analysis

Data platforms have emerged as ‘pulley mechanisms’ that enable research collaborations that are cost-effective and efficient, making better use of limited existing data and the scarce economic resources that are available. These platforms facilitate the pooling and harmonisation of data sets from multiple centres or investigations, thereby allowing data reuse and amplifying statistical power by enabling individual patient data meta-analysis (IPD-MA). In an IPD-MA, deidentified patient-level data sets are pooled, variables are mapped to common definitions and analyses can be run across the combined data set. The topic of which types of data are hosted on a data platform, and the process for how data are contributed to and accessed from the platform, has been well documented and generally involves collecting, harmonising and hosting IPD from clinical trials (for further details, see^17^,^19^).

When adequate IPD exist, though dispersed across studies, IPD-MA can generate new scientific evidence from the IPD pooling process which cannot be extracted from existing publications or produced from typical aggregated meta-analyses. Because an IPD-MA uses patient-level records, it can standardise, when possible, the definitions of the outcome, exposure and covariates across studies and test effect modification. Compared with a meta-analysis of aggregated data from individual studies, an IPD-MA typically yields more robust and reliable evidence, particularly when looking at dosing-specific recommendations for specific subgroups.^17 20 21^ In addition, many paediatric clinical trials are either discontinued or unpublished,^22^ rendering their (aggregated) results unavailable for synthesis in aggregated data meta-analyses. An IPD-MA can, very economically, make use of the IPD collected in these trials even when it is unlikely that the individual trial results would be analysed and published.

An IPD-MA can, in specific circumstances, also produce evidence which would otherwise require conducting prospective studies.^23^ It is less time-consuming than conducting new clinical trials, is less costly, and allows for quicker dissemination of findings and more timely evidence-based adjustments to healthcare policy and clinical practice guidelines. Our experience is that conducting an IPD-MA can be an order of magnitude less costly way to address the same research question compared with primary data collection, although more precise estimates will be generated in future research currently under preparation. Expanding robust data platforms, to enable more IPD-MA, could therefore serve as a valuable strategy to address the underprovision of essential drug research for specific subpopulations, particularly paediatric populations in LMICs.^3^ In addition, research on vulnerable populations must adhere to ethical principles; maximising the value of already existing clinical data in an IPD-MA before resorting to new primary data collection might be protective of such populations.^7 24^

Performing IPD-MAs in practice presents many challenges and limitations. IPD-MAs are often limited by the availability of and access to the underlying IPD, as well as by governance constraints around privacy, consent, pseudonymisation and data protection, especially for legacy paediatric trials. They also require substantial effort to harmonise exposures, outcomes, covariates and laboratory measurements across studies, with residual heterogeneity sometimes remaining even after standardisation. Their external validity depends on how representative the contributing studies are of broader LMIC paediatric populations. Finally, IPD-MAs can be more logistically and financially demanding than meta-analyses of aggregated data, and this requires sustained stakeholder engagement, secure and compliant data governance, and fair attribution of credit to collaborating research teams.

These limitations suggest that IPD-MAs should be deployed discriminately in settings where the required data exist and the costs to access and analyse these data can be supported. If data sharing becomes a complementary component of regular clinical trial activities and data platforms are in place to support data access, curation and harmonisation, the incremental financial, time and logistical demands of IPD-MA should fall. By harnessing economies of scale and scope, data platforms can reduce the burden borne by any single IPD-MA, while also addressing challenges around data governance and supporting equitable partnerships with data contributors from LMICs.

## The role of data platforms as ‘pulley mechanisms’: WWARN case study

The WorldWide Antimalarial Resistance Network (WWARN)—a malaria-focused research platform hosted within the Infectious Diseases Data Observatory (IDDO) at the University of Oxford—exemplifies the practical utility of the data sharing approach.^17^ Since its inception in 2009, WWARN’s data platform has facilitated IPD-MAs of the safety, tolerability and efficacy of a number of antimalarial drugs.^18 25^ Collaborating researchers can add their IPD to the data repository for subsequent use and reuse in IPD-MAs, maximising the value that can be extracted from their data in a cost-efficient manner. Studies that used IPD hosted on WWARN’s data repository have gone on to inform WHO guidelines for the treatment of malaria in paediatric populations.^19^ For example, a 2013 IPD-MA by a WWARN Study Group evaluated the impact of dosing regimens on the antimalarial efficacy of dihydroartemisinin–piperaquine. The results contributed to revision of the WHO treatment guidelines starting in 2015 for children with malaria.^17 26 27^ In a recent IPD-MA, WWARN demonstrated the lower efficacy and post-treatment prophylaxis effect of ACTs in acutely malnourished children younger than 5 years, a subpopulation systematically underrepresented in clinical trials^28^ but sufficiently numerous once their IPD was gathered in an IPD-MA. This result contrasted with inconsistent findings on the effects of malnutrition on malaria treatment response reported in the literature.^29^ The benefits of guideline changes or generating new scientific evidence to benefit global public health—and children in particular—are a direct consequence of WWARN’s data reuse model, as it would not have been cost-effective to generate this evidence through conventional means such as industry- or investigator-initiated clinical trials. Importantly, in addition to serving as a cost-efficient catalyst for IPD-MA-style research designs, WWARN ensures equitable and fair attribution to data contributors, most of whom are located in LMICs, thereby promoting the fairness and sustainability of the model.

## Conclusion

Maximising value-for-money from available resources is especially crucial for neglected or rare diseases and diseases which primarily affect resource-poor populations. In these contexts, commercial incentives for industry to fund large-scale de novo clinical trials are typically weak and the resources available to study restricted subpopulations such as children are particularly limited.^1^ Investing in data platforms can serve as a ‘pulley mechanism’ to enable IPD-MA of data from disparate trials, including those that did not generate reportable findings. The success of data platforms like IDDO demonstrates the potential of this model as an enabler of more powerful evidence development and as an efficient economic model for research that improves evidence-based healthcare in LMICs. Along with improved evidence and more efficient use of data, complementary efforts are needed to ensure that drugs are affordable and available in formulations that allow for the right dose to be administered in the right form to the right patient at the right time. While the impact of data platforms may be greatest for children in LMICs due to the commercial factors at play, global efforts to share, pool, harmonise and analyse IPD of both children and adults should be welcomed as a cost-effective step towards improving global public health.
