# Supplementary material for: Individual patient data meta-analysis: a cost-effective and efficient tool to advance paediatric research in low- and middle-income countries
Source: BMJ Open. 2026 May 13;16(5):e100150. doi: 10.1136/bmjopen-2025-100150 (PMC13182405; doi:10.1136/bmjopen-2025-100150)
Supplement: Supplementary file 1 [file bmjopen-2025-100150.reviewer_comments.pdf]

PEER REVIEW HISTORY

BMJ Open publishes all reviews undertaken for accepted manuscripts. Reviewers are asked to complete a checklist review form and are provided with free text boxes to elaborate on their assessment. These free text comments are reproduced below.

ARTICLE DETAILS

Title (Provisional)

Individual patient data meta-analysis: a cost-effective and efficient tool to advance paediatric research in low- and middle-income countries

Authors

McElwee, Frederick; Farlow, Andrew; Guerin, Philippe

VERSION 1 - REVIEW

**Reviewer**	**1**
**Name**	**Holloway , Adrian**
**Affiliation**	**University of Maryland Medical Center, Pediatrics**
**Date**	**24-Oct-2025**
**COI**	**None**

It is my pleasure to review this manuscript describing the potential benefits of IPD to improve and advance research in LMIC. The authors, McElwee et al, provide an interesting and compelling argument to help mobilize already available data to improve medication delivery and development for neglected tropical diseases. As a global health researcher, I personally appreciate the acknowledgement that neglected does not mean that the impact or burden is low. I have minor edits/points of clarification with regard to this proposed manuscript:

1. In the section describing the economic impediments to pediatric R&D, could the authors describe specific examples of the failure to economic reward to companies in LMIC? Writ large... this is one of the pillars of lack of development in LMIC, but specific example or two would be impactful.

2. I think the argument for IDP would be served by addressing challenges and limitations in performing/obtaining IDP especially concerning access to unpublished data, accessing proprietary respositories, and challenges in power asymmetry with regards to ownership and publication of data versus access for those in LMIC.

**Reviewer**	**2**

**Name**	**Hashmi, S. Shahrukh**
**Affiliation**	**University of Texas Health Science Center Houston**
**Date**	**25-Nov-2025**
**COI**	**None**

This is a well-written manuscript detailing the promise of using IPD-MA as a cost-effective research paradigm to facilitate research benefiting children in LMICs. The authors have nicely detailed the current barriers to research for rare diseases or those primarily affecting resource-poor populations. In addition, they have described the "pulley mechanism" to facilitate research and the role IPD-MAs can play. I agree with the authors that the "pulley mechanism" paradigm, and IPD-MAs in specific, are extremely promising options in promoting pediatric research in LMICs. However, the authors have not said much about the challenges associated with IPD-MAs and collation of data in large data platforms.

For the benefit of readers who may not be as well-versed in IPD-MAs, I would recommend including some additional information to give a more holistic review of this analytic technique. The recommendations can be grouped into two main categories: 1) describing challenges and 2) describing the "cost-efficiency"

1) Some challenges to consider could be:

a. data availability and accessibility issues (formats, ownership, etc.)

b. data sharing concerns (including privacy and consent issues, anonymization, etc.)

c. heterogeneity in definitions and measurements (need to standardize population, clinical and laboratory data, etc.)

d. logistical burdens related to coordination and collaboration (motivation of stakeholders, long-term engagement, etc.)

e. cost related to data storage and management (standardized workflows, how are cost burdens shared, etc.)

f. representativeness and generalizability of data (dependent on sources of data, demographics, external social/economic/healthcare factors)

2) Regarding "cost efficiency", perhaps the authors can provide:

a) data to support the claim of this being an "efficient economic model" to increase evidence-based data in LMICs

b) information on how the higher costs of IPD-MAs compared to traditional meta-analyses is balanced by the power of harmonizing fragmented datasets and avoiding expensive primary research

For each of these two categories, I would recommend brief, albeit sufficient, information to provide a well-rounded view of IPD-MAs.

VERSION 1 - AUTHOR RESPONSE

Reviewer 1:

Comment: 1. In the section describing the economic impediments to pediatric R&D, could the authors describe specific examples of the failure to economic reward to companies in LMIC? Writ large... this is one of the pillars of lack of development in LMIC, but specific example or two would be impactful.

Response: Given the brevity of the space allowed, we introduce in this section two antimalarials where these commercial impediments impeded pediatric R&D, dihydroartemisinin-piperaquine and artemisinin-based combination therapies (ACTs) for children, noting in particular that even if children might benefit from a better dosing regimen, it would generate no financial reward for a company and not justify to them the costs of a clinical trial. We then link back to this failure when we cover the two cases further below. Later we now mention several high-priority areas, where commercial incentives are very week and trials hard to justify, needing paediatric oral dosage forms, acoziborole for human African trypanosomiasis (HAT) and miltefosine and amphotericin B for visceral leishmaniasis (VL)

Comment: 2. I think the argument for IDP would be served by addressing challenges and limitations in performing/obtaining IDP especially concerning access to unpublished data, accessing proprietary respositories, and challenges in power asymmetry with regards to ownership and publication of data versus access for those in LMIC.

Response: We agree with you that we need to be more nuanced about the benefits but also challenges of performing IDP. We have added a discussion of the challenges to the section on the “Role of Data Platforms as Pully Mechanisms”. We also then add how this suggests a complementary role for IDP alongside clinical trial activities. Regarding unpublished data and proprietary repositories, we note that systems governing unpublished data and proprietary repositories may need to be changed, as an extension of the not-so-long-ago innovation that all clinical trials be registered, and we finish on an argument for tackling the asymmetries mentioned. We hope this achieves the critical balance you spotted was missing.

Reviewer: 2

Comment: This is a well-written manuscript detailing the promise of using IPD-MA as a cost-effective research paradigm to facilitate research benefiting children in LMICs. The authors have nicely detailed the current barriers to research for rare diseases or those primarily affecting resource-poor populations. In addition, they have described the "pulley mechanism" to facilitate research and the role IPD-MAs can play. I agree with the authors that the "pulley mechanism" paradigm, and IPD-MAs in specific, are extremely promising options in promoting pediatric research in LMICs. However, the authors have not said much about the challenges associated with IPD-MAs and collation of data in large data platforms.

Response: Thank you very much for your very supportive language. We are glad you thought our manuscript was well written and nicely details the barriers to current research. We are glad that you too can see the promise of IPD-MAs. We agree that we did not nuance the argument enough with reference to the challenges of IPD-MAs. We have greatly changed the section “Role of Data Platforms as Pully Mechanisms” to tackle the challenges you mention.

Comment: For the benefit of readers who may not be as well-versed in IPD-MAs, I would recommend including some additional information to give a more holistic review of this analytic technique. The recommendations can be grouped into two main categories: 1) describing challenges and 2) describing the "cost-efficiency"

1) Some challenges to consider could be:

a. data availability and accessibility issues (formats, ownership, etc.)

b. data sharing concerns (including privacy and consent issues, anonymization, etc.)

c. heterogeneity in definitions and measurements (need to standardize population, clinical and laboratory data, etc.)

d. logistical burdens related to coordination and collaboration (motivation of stakeholders, long-term engagement, etc.)

e. cost related to data storage and management (standardized workflows, how are cost burdens shared, etc.)

f. representativeness and generalizability of data (dependent on sources of data, demographics, external social/economic/healthcare factors)

Response: We have tried to cover all of these in the section “Role of Data Platforms as Pully Mechanisms”. As noted, if IPD-MAs are to move from being ad-hoc case-by-case deployments to being a part of a collective strategy to extract the maximum value from extant clinical data, some issues need collective effort which we can but hint at here.

Comment: 2) Regarding "cost efficiency", perhaps the authors can provide:

a) data to support the claim of this being an "efficient economic model" to increase evidence-based data in LMICs

b) information on how the higher costs of IPD-MAs compared to traditional meta-analyses is balanced by the power of harmonizing fragmented datasets and avoiding expensive primary research

Response: We agree with the need to develop evidence for the relative cost and cost-effectiveness of IPD-MAs compared to traditional meta-analyses and to primary research. A detailed cost and cost-effectiveness analysis will be reported in a separate, future research publication under preparation, and our intention was to keep the scope of this communication article on the idea that IPD-MAs can enable research in neglected areas, such as diseases affecting paediatric populations in LMICs, given the economic challenges that are typically associated with this type of research. To address the reviewer's points, we have strengthened the text in the section "Role of Data Platforms as Pully Mechanisms". To address point a), we have added the following: "Our experience is that conducting an IPD-MA can be an order of magnitude less costly to address the same research question compared with primary data collection, although more precise estimates will be generated in future research currently under preparation." To address point b), we have added: "Compared with meta-analyses of aggregated data from individual studies, IPD-MAs typically yield more robust and reliable evidence (16–18). However, IPD-MAs can be costlier both in terms of time and the resources required."

VERSION 2 - REVIEW

**Reviewer**	**1**
**Name**	**Holloway , Adrian**
**Affiliation**	**University of Maryland Medical Center, Pediatrics**
**Date**	**12-Feb-2026**
**COI**	

I appreciate the interesting and innovative approach to using IPD-MA for pediatric indications for drug development, framing the problem as an imbalance between financial incentive and burden of disease, and examples of how this method has been successful. I think the authors should spend some space investing more detail into what IPD-MA is and how it is superior (for the purposed outlined in the manuscript) superior to meta-analyses. The authors to a fantastic job describing the impediments to R&D but short the reader on the potential uses and benefits of IPD-MA before going into the WWARN example. Space is short, however, making some of the other sections shorter will allow room for that focus. There is a bit of reduncancy throughout; I have noted the examples in my review. When referring to cost benefits, there is potential for confusion. The authors state simultaneously that it can be more expensive BUT cheaper throughout; if I have understood the manuscript well--the implication that initial costs may be higher due to data platforming but cheaper overall than starting a new study. If the authors are going to discuss data platforming and harmonization as an essential component of this--more clarity about what that looks like, what the process is, and what KINDS of data should be included.

Please see my detailed review and comments on the attached manuscript.

**** The reviewer provided a marked copy with additional comments. Please contact the publisher for full details. ****

**Reviewer**	**2**
**Name**	**Hashmi, S. Shahrukh**
**Affiliation**	**University of Texas Health Science Center Houston**
**Date**	**02-Feb-2026**
**COI**	

The authors have adequately responded to the reviewer comments and have addressed the concerns raised. The edits and additions to the manuscript have definitely improved the robustness of the report and provide a more balanced view and promise of IPD-MAs.

The authors mentioned in their response to the reviewer that certain aspects such as more detailed information on cost-effectiveness has not been added due to the level of detail required (that the current format does not permit) and a separate research publication being prepared to specifically tackle that. In light of that, the information they have provided is adequate for this current manuscript.

VERSION 2 - AUTHOR RESPONSE

Response to reviewers:

We would like to thank both reviewers for their thorough and constructive feedback, which has significantly strengthened and improved the manuscript.

Reviewer: 1 [PLEASE SEE ATTACHED FILE FOR ADDITIONAL COMMENTS FROM REVIEWER 1 AS COMMENTS ON THE MANUSCRIPT]
Dr. Adrian Holloway, University of Maryland Medical Center
Comments to the Authors:
I appreciate the interesting and innovative approach to using IPD-MA for pediatric indications for drug development, framing the problem as an imbalance between financial incentive and burden of disease, and examples of how this method has been successful. I think the authors should spend some space investing more detail into what IPD-MA is and how it is superior (for the purposed outlined in the manuscript) superior to meta-analyses. The authors to a fantastic job describing the impediments to R&D but short the reader on the potential uses and benefits of IPD-MA before going into the WWARN example. Space is short, however, making some of the other sections shorter will allow room for that focus.

There is a bit of redundancy throughout; I have noted the examples in my review (ATTACHED).

- We greatly appreciate the feedback and attached comments. We address points made as comments on the document point-by-point in the attached file (tracked version).

- We have added a more comprehensive description of the statistical advantages of IPD-MA in the section ‘The Role of Data Platforms…’.
- We have made an effort to remove text that is redundant or unnecessary. The reviewer’s comments were really helpful in enabling us spot this and write more succinctly. Thank you.

When referring to cost benefits, there is potential for confusion. The authors state simultaneously that it can be more expensive BUT cheaper throughout; if I have understood the manuscript well--the implication that initial costs may be higher due to data platforming but cheaper overall than starting a new study. If the authors are going to discuss data platforming and harmonization as an essential component of this--more clarity about what that looks like, what the process is, and what KINDS of data should be included.

Please see my detailed review and comments on the attached manuscript.

- We appreciate this comment and have aimed to clarify in what context IPD-MA is more expensive and in what context it is cheaper; namely, IPD-MA is more expensive than aggregated-data meta-analysis, but cheaper than the cost of new clinical trials. Again, it was really useful for the reviewer to help us spot this and articulate it better to the reader.
- We are not arguing that data platforming is essential to IPD-MA – indeed, many IPD-MAs have been conducted without accessing data held on a data platform. Instead, we are stating that the cost of IPD-MA can be significantly reduced, and results speeded up, if data platforms are in place to allow researchers to access centralised and potentially harmonised IPD.
- In the section “The Role of Data Platforms…”, we have clarified that data platforms should host ‘de-identified patient-level datasets’ and we briefly comment on the process for platforming IPD. Given the positioning of this article as a ‘communication article’, we provide a high-level summary and direct readers to further resources on this topic for more detail.

Reviewer: 2
Dr. S. Shahrukh Hashmi, University of Texas Health Science Center Houston
Comments to the Authors:
The authors have adequately responded to the reviewer comments and have addressed the concerns raised. The edits and additions to the manuscript have definitely improved the robustness of the report and provide a more balanced view and promise of IPD-MAs.

The authors mentioned in their response to the reviewer that certain aspects such as more detailed information on cost-effectiveness has not been added due to the level of detail required (that the current format does not permit) and a separate research publication being prepared to specifically tackle that. In light of that, the information they have provided is adequate for this current manuscript.

Thank you for your prior feedback and supportive comments.

VERSION 3 - REVIEW

**Reviewer**	**1**
**Name**	**Holloway , Adrian**
**Affiliation**	**University of Maryland Medical Center, Pediatrics**
**Date**	**21-Apr-2026**
**COI**	

This is extremely well done and addresses my concerns. This is an interesting field of research methodology and would like to see how this can be operationalized in the future.
